# A meta-analysis of the prevalence of *Salmonella* in food animals in Ethiopia

**DOI:** 10.1186/s12866-014-0270-y

**Published:** 2014-11-15

**Authors:** Getachew Tadesse, Tesfaye S Tessema

**Affiliations:** Department of Biomedical Sciences, College of Veterinary Medicine and Agriculture, Addis Ababa University, P.O. Box 34, Debra Zeit, Ethiopia; Institute of Biotechnology, College of Natural and Computational Sciences, Addis Ababa University, P.O. Box 1176, Addis Ababa, Ethiopia

**Keywords:** Animals, Ethiopia, Prevalence, *Salmonella*, Serotypes

## Abstract

**Background:**

The globalization of the food supply and the increased movements of people, animals and goods have increased the threat of *Salmonella* infections in several countries. The objective of this study was to estimate the prevalence of *Salmonella* in food animals in Ethiopia by using meta-analytical methods.

**Results:**

The prevalence of *Salmonella* in slaughtered cattle, sheep, goats and pigs were 7.07%, 8.41%, 9.01% and 43.81% respectively. The occurrence of *Salmonella* was significantly higher in pigs than in slaughtered true ruminants (p <0.001) but not significantly different between cattle, sheep and goats (*p* >0. 05). *S.* Mishmarhaemek, *S.* Infantis and *S.* Hadar were the predominant isolates in cattle, small ruminants and pigs respectively. *S.* Typhimurium was isolated from all host species.

**Conclusions:**

All food animals are considerable reservoirs of *Salmonella* and pose a significant risk to public health*.* Safety measures in slaughter houses and butcheries and education of the public could reduce the risk of transmission of *Salmonella* from animals to humans.

**Electronic supplementary material:**

The online version of this article (doi:10.1186/s12866-014-0270-y) contains supplementary material, which is available to authorized users.

## Background

Globally, non-typhoidal *Salmonella* (NTS) is a cause of about 155, 000 human deaths each year [1] and the threat of epidemic infections has increased due to the globalization of the food supply and the increased movements of people, animals and goods within and between countries [2,3]. Apart from the morbidity and mortality costs in humans and animals, restrictions to trade and discard of contaminated food are important socioeconomic problems of the bacteria [4].

Humans acquire infection through the consumption of contaminated products or contact with infected animals [5-10]. *S.* Typhimurium and *S.* Enteritidis are common causes of human diseases [11] and nowadays, the spread of multidrug resistant (MDR) serotypes has become a global concern. For instance, since its description in 2006, in Ethiopia [12], a highly MDR *S*. Kentucky strain has been isolated from domestic and wild animals and humans in Africa, Europe and Asia [13]. Similarly, MDR *S*. Concord was isolated from Ethiopian adoptees in Europe and the USA [14,15] and a highly invasive *S*. Typhimurium strain (ST313) has occupied a niche provided by HIV, malaria, and malnutrition in Africa [16]

Ethiopia has the largest animal population in Africa and the living standard of the population is generally favorable for the transmission of pathogens from animals to humans and the vice versa. Despite Salmonellosis being one of the important zoonotic diseases, surveillance and monitoring systems are not in place and the temporal and spatial distributions of the serotypes are not described. The objective of this study was to estimate the prevalence of *Salmonella* in food animals by using meta-analytical methods.

## Methods

The guideline of the PRISMA group (Preferred Reporting Items for Systematic Reviews and Meta-Analyses) [17] was followed in the reviewing and the check list was used to ensure inclusion of relevant information (see Additional file 1). An animal was considered to be a carrier if *Salmonella* was detected in the mesenteric lymph nodes (MLN) and/or the gastro-intestinal tract (GIT) contents. A serotype was considered to be dominant if it accounted for more than 5% of the serotyped isolates in each host group.

### Search and selection of studies

The search strategy was described in a previous study [18]. Briefly, studies were searched in Medline, Google scholar and the lists of references of articles. The last search was done on September 27, 2014. Eligible studies were selected by using inclusion and exclusion criteria. A study was eligible if it (a) was published in English, (b) was cross sectional, (c) was on apparently healthy animals and (e) described the study design and microbiological methods. Studies with titles and abstracts that were not relevant to the outcomes of interest and studies that did not meet the eligibility criteria or with inappropriate data were excluded.

### Data abstraction

From each eligible study, the first author, year of publication, year of study, location, host species, sampling design, number of animals, microbiological methods, number of *Salmonella* positive MLN and GIT (small intestinal/caecal/ fecal) content samples were extracted. The study level prevalence (p) and standard error (s.e) were calculated by the following formulae: p = np/n and s. e. =√ p (1-p*)*/n: where np = number of positive samples and n = number of samples. The data was extracted by TG.

### Data analysis

To produce conservative estimates, a zero reported for the numbers of positive samples was imputed as 0.5 [19]. To normalize the data, the study level estimates were transformed to logit event estimates [20,21]: lp = ln [p/ (1 − p)], where lp = the logit event estimate; ln = the natural logarithm; p = study level estimate. The variance of the logit event estimates was calculated by the following formula: v (lp) =1/ (np) +1/ [n (1 − p)], where v = variance and n = sample size. The data were grouped and analyzed as ruminant and non-ruminant data. A subgroup analysis was done by species of ruminants.

### Risks of bias and heterogeneity

The qualities of the sampling design and the microbiological methods were used to assess the within study biases. A funnel plot was used to get a visual impression of the across study bias (small study effects) of the study level estimates in ruminants. The statistical significance of the bias was assessed by the Egger’s regression asymmetry test [22]. The Duval and Tweedie nonparametric ‘trim and fill’ linear random method was used to calculate unbiased estimates [23].

The heterogeneity of the estimates in ruminants was visually examined by the Galbraith plot [24]. The statistical significance of the heterogeneity was assessed by the Cochran’s Q test and a non significant heterogeneity was accepted if the ratio of Q and the degree of freedom (Q/df) was less than one. The inverse variance index (I^2^) was used to quantify the percentage of the variation in prevalence estimates attributable to heterogeneity. I^2^ values of 25%, 50% and 75% were considered as low, moderate and high heterogeneity respectively [25].

### Pooled estimates

The DerSimonian and Laird random effects model [26] was used to pool logit event estimates. The pooled logit estimates were back transformed to prevalence estimates (p) by the following formula: p = e^lp^/(e^lp^ + 1): where e = the base of the natural logarithm. The sensitivity of the pooled prevalence of *Salmonella* in ruminants was assessed by single study omitted influence analyses. Whether a pooled estimate is significantly different from zero or not was tested by the Z test. A study was considered to be influential if the pooled estimate without it was not within the 95% confidence limits of the overall mean. The Yates corrected Chi Square test was used to test the significance of the differences between pooled estimates [27,28]. Alpha was set at 0.05.

Microsoft Office Excel 2007 was used to calculate study level prevalence estimates, logit event estimates and standard errors and to transform logit event estimates to prevalence estimates. Epi info™ (Version 3.5.1, Center for Disease Control, CDC, USA) was used to compare groups. Stata (Version 11.1, Stata Corp, College Station, Texas) was used in all other analyses.

## Results and discussion

### Search results and eligible studies

**Figure 1:**
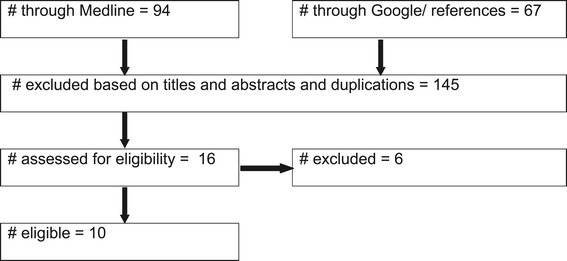
**A flow diagram of the selection of studies.**

Figure 1 shows the literature search results. The search yielded 161 reports. One hundred and forty five reports were excluded because the titles and abstracts were not relevant to the outcomes of interests. Of the screened articles, six were excluded due to sampling design, data inconsistency, pooled sample examination, lack of separate information on the number of samples taken from a slaughtering plant and markets, apparently sick and dead animals and small sample size. A total of 10 studies were eligible for quantitative syntheses [29-38].

### Characteristics of the eligible studies

Table 1 depicts the characteristics of the eligible studies. The studies were conducted between 1999 and 2010 in central, Northern and Eastern Ethiopia during the dry and short rainy seasons (October through May). Nine studies were on slaughtered animals and one was on dairy cattle. Whilst most slaughtered ruminants were derived from the extensive production systems in the rural areas, dairy cattle and pigs were from semi-intensive/intensive production systems. A total 3435 MLN and intestinal content samples from 1815 animals (119 camels, 220 goats, 293 sheep, 379 pigs and 804 cattle) were examined to detect *Salmonella.* The study level estimates ranged from 1.9% in cattle to 45.5% pigs.

**Table 1:** **Characteristic of the eligible studies**

**Author**	**Host**	**Sy**	**Lo**	**n**	**OA**	**GIT**	**MLN**
					**p (%)**	**p (%)**	**p (%)**
[29]	Cattle	1999/2000	DZ	323	6 (1.9)	2 (0.62)^a^	4 (1.2)
[30]	Cattle	2005/6	DZ	100	14 (14)	6 (6)^b^	8 (8)
[31]	Cattle	2006/7	BD	186	13 (7)	11(5.9)^c^	6 (3.2)
[32]	Cattle	2010	AA	195	21 (10.8)	15 (7.7)^a^	-
[33]	Sheep	2002/3	DZ	47	1 (2.1)	1 (2.1)^a^	0 (0)
[34]	Sheep	2003/4	AM	104	12 (11.5)	5 (4.8)^a^	8 (7.7)
[35]	Sheep	2007/8	MJ	142	11 (7.8)	3 (2.1) ^b^	8 (5.6)
[33]	Goats	2002/3	DZ	60	9 (15)	2 (3.3)^a^	7 (11.7)
[34]	Goats	2003/4	AM	100	3 (3)	2 (2)^a^	2 (2)
[35]	Goats	2007/8	MJ	60	7 (11.7)	4 (6.7)^b^	3 (5)
[36]	Pigs	2004/5	AA	278	120 (43.2)	63 (22.7)^b^	99 (35.6)
[37]	Pigs	2004/5	DZ	101	46 (45.5)	17 (16.8)^b^	42 (41.6)
[38]	Camels	2001/2	DJ	119	28 (23.5)	18 (15.13)^a^	19 (15.9)

AA, Addis Ababa; AM, Addis Ababa and Modjo; BD, Bahirdar; DZ, Debrezeit; DJ, Diredawa and Jijiga; GIT, gastrointestinal tract; Lo, location; MJ, Modjo; MLN, mesenteric lymph nodes; n, number of animals; p, number of positive animals; OA, overall animal; Sy, study year.

^a^Faeces.

^b^Caecal contents.

^c^Small intestinal contents.

### Risks of bias and heterogeneity

Sampling was random in seven studies [30-35,37] and all animals presented for slaughter in each sampling day were sampled in three studies [29,36,38]. The analytical units were 25 g MLN and GIT contents in eight studies [29-31,33,34,36-38], one gram of feces and one milliliter of milk in a study on dairy cattle [32] but not reported in one study [35]. In all studies *Salmonella* was isolated and identified according to the guideline of the International Organization for Standardization (ISO 6579, 1998-2002) with some modifications. Both the funnel plot and the Egger’s regression asymmetry test did not suggest the presence of bias and the Duval and Tweedie nonparametric method did not incorporate theoretical missing studies. Accordingly, the within and across study biases were considered negligible.

**Figure 2:**
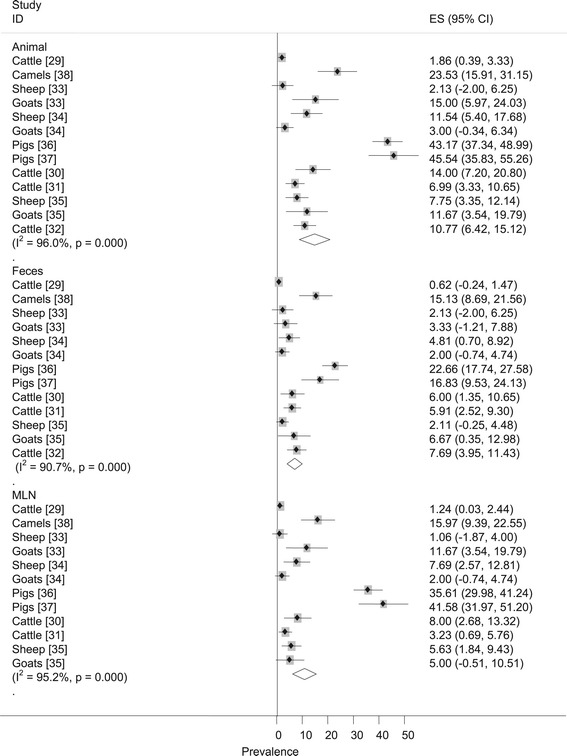
**Forest plots of the prevalence of** ***Salmonella*** **.**

Figure 2 presents forest plots of the untransformed prevalence estimates. The I^2^ values of the logit event estimates in ruminants and pigs were 81.3% and Zero respectively. In a subgroup analysis of the ruminant data by host species, the I^2^ was 39.9% in sheep, 69.4% in goats and 89.1% in slaughtered cattle (Table 2). The moderate to high heterogeneities could be due to several factors including origin of animals, breed, management and exposure to stress. However, the effects of all potential factors but species could not be put in context because of the absence of data and the limited number of studies in further sub groupings.

**Table 2:** **Pooled prevalence of** ***Salmonella*** **by host species**

**Host species**	**Pooled estimate**		**Heterogeneity**		
	**p (95% CI)**	***Z-p***	**I** ^**2**^	**Q-** ***p***	**Q/df**
Ruminants^†^	7.47 (4.75,11. 58)	0.000	73.4	0.000	3.76
Cattle	7.07 (2.05,16.17)	0.000	89.1	0.000	9.17
Sheep	8.41 (4.77,14.42)	0.000	39.9	0.189	1.67
Goats	9.01 (3.88, 19.62)	0.000	69.4	0.038	3.27
Pigs	43.81 (38.89,48.85)	0.000	0.0	0.680	0.09

df, degrees of freedom; I^2^, Inverse variance index; Q-*p*, probability value of Cochran’s Q test; Z-*p,* probability value of Z test.

^†^Slaughtered ruminants; all single study omitted pooled estimates were within the 95% confidence limits of the overall mean.

### Pooled prevalence

The prevalence of *Salmonella* in slaughtered cattle, sheep, goats and pigs were 7.07%, 8.41%, 9.01%, and 43.81% respectively (Table 2). Pooled estimates for camels and dairy cattle were not calculated because each had single reports. Comparison of the estimates with reports elsewhere in Africa is difficult because reports based on national surveys or meta-analytical studies are scarce. However, national survey reports from developed countries show lower fecal/caecal estimates in cattle (0.2%-6.8%) [39-42], sheep (0.1%) [39] and pigs (2.5%-23%) [39,40,43-46]. In addition, the proportions of *Salmonella* positive swine production holdings in Europe range from zero in Bulgaria, Sweden, Finland and Norway to 30% in UK [47]. In Denmark, since the initiation of the control program in 1988/89, the prevalence of *Salmonella* in poultry and swine has markedly reduced [48,49]. In general, there is no set standard and differences across countries could be attributed to several factors that may involve animal, environmental and management factors, control measures and the study methods.

Tables 3 shows pooled prevalence of *Salmonella* in the MLN and GIT contents by host species. The estimates depict the level of infection in animals and their potential to contaminate animal products, humans, animals and the environment. The relation between humans and animals in Ethiopia is so close to such an extent of sharing the same roof and animal wastes are not properly disposed off. Moreover, the meat handling practice in slaughter houses and butcheries is generally unhygienic [50-52] and backyard slaughtering and raw meat and milk consumption are wide spread practices. In general, the unhygienic living circumstances and lack of awareness of the population on zoonotic diseases are suggestive of the considerable risk associated with the transmission of *Salmonella* from animals to humans. Elsewhere, outbreaks through contact with chicks and livestock were reported [53,54].

**Table 3:** **Pooled prevalence of** ***Salmonella*** **in the MLN and GIT contents**

**Host species**	**Mesenteric lymph node**					**GIT contents**				
	**p (95% CI)**	***Z-p***	**I** ^**2**^	***Q-p***	**Q/df**	**p (95% CI)**	***Z-p***	**I** ^**2**^	***Q-p***	**Q/df**
Ruminants^†^	4.7 (2.88,7.57)	0.000	61.8	0.007	2.62	3.66 (2.31, 5.74)	0.000	42.5	0.084	1.74
Cattle	3.34 (1.17,9.19)	0.000	79.9	0.007	4.98	3.35 (1.11,9.58)	0.000	78.9	0.009	4.75
Sheep	6.19 (3.77,10)	0.000	5.0	0.349	1.06	3.33 (1.74,6.28)	0.000	0.0	0.462	0.78
Goats	5.56 (1.99,14.600)	0.000	64.6	0.059	2.83	4.08 (1.99,8.17)	0.000	6,7	0.342	1.07
Pigs	37.38 (32.24, 42.85)	0.000	11.4	0.258	1.13	20.67 (15.68, 26.76)	0.000	33.4	0.221	1.5

df, degrees of freedom; I^2^, Inverse variance index; Q-*p*, probability value of Cochran’s Q test; Z-*p,* probability value of Z test.

^†^Slaughtered ruminants; all single study omitted pooled estimates were within the 95% confidence limits of the overall mean.

The prevalence of *Salmonella* was higher in pigs than in slaughtered ruminants [X^2^ = 266.5; p <0.001 (OR = 9.63 (95% CI = 7.05, 13.17)] but not significantly different between cattle, sheep and goats (p >0.05). The occurrence of *Salmonella* in the MLN was higher in pigs than in slaughtered ruminants [X^2^ = 172.32; p <0.001; OR = 11.98 (95%CI = 7.67, 18.81)] but not affected by species of slaughtered true ruminants (p >0.05). The occurrence of *Salmonella* in the caecal contents of pigs was significantly higher than the estimate for slaughtered ruminants [X^2^ = 72.08; p <0.001; OR = 6.91 (95%CI = 4.12, 11.68)] but not among species of slaughtered true ruminants (p >0.05). The higher occurrence of *Salmonella* in pigs compared to ruminants is apparently due to the coprophagous feeding behavior and higher exposure of the former to contaminated sources. Therefore, pork is more likely to be contaminated compared to beef, mutton or goat meat and individuals routinely or occupationally exposed to pigs are at a higher risk of acquiring *Salmonella* than individuals in contact with true ruminants. Similarly, despite a few reports, the risk of acquiring S*almonella* from dairy cattle and camels appears higher than the risk from beef cattle, sheep and goats.

### Dominant serotypes

Table 4 depicts the dominant serotypes. Of the 582 Salmonella isolates, 507 were serotyped. Twenty one isolates of dairy cattle [32] and 54 isolates of small ruminants [35] were not serotyped. The numbers of serotypes identified from slaughtered pigs, camel, cattle, sheep and goats were 28, 15, 15, 11 and 10 respectively. The dominant serotypes isolated from cattle, small ruminants, pigs and camels accounted for 72.46%, 67.27%, 73.78% and 81.04% of the isolates in each group respectively. These serotypes were reported in two or more studies on animals or animal products or humans in Ethiopia: *S*. Anatum [30,55-60], *S*. Braenderup [33,38,56-59], *S*. Hadar [33,36,37,56,59], *S*. Havana [36-38], *S*. Butantan [30,38,61], *S*. Heidelberg [31,34,38,62], *S*. Kentucky [33,36,37,59], *S*. Kottbus [36,56,59,62], *S*. Mishmarhaemek [29,31], *S*. Muenchen [36,38,60,62], *S*. Newport [30,31,37,59], *S*. Saintpaul [36,38,55,57-59] and *S*. Typhimurium [29-31,33,34,36-38,56,57,59-62]. The occurrences of the serovars in different samples suggest their wide distribution across several animal populations and regions in Ethiopia. However, the relative preponderances and distributions of the serovars could differ by agro-climatic zones.

**Table 4:** **Frequencies (%) of dominant serotypes**

**Host, n, Authors**	**Serotype**	**Number (%)**
Cattle, (n = 69), [29-31]^‡^	*S*. Mishmarhaemek	14 (20.3)
	*S*. Typhimurium	12 (17.4)
	*S*. Newport	9 (13)
	*S*. Eastbourne	6 (8.7)
	*S*. Infantis	5 (7.3)
	*S*. Anatum	4 (5.8)
	Others	19 (27.5)
Small ruminants, (n = 55), [33,34]	*S*. Infantis	15 (27.3)
	*S*. Typhimurium	10 (18.2)
	*S*. Butantan	8 (14.6)
	*S*. Heidelberg	4 (7.3)
	Others	18 (32.7)
Pigs, (n = 267), [36,37]	*S.* Hadar	85 (31.8)
	*S*. Eastbourne	40 (15)
	*S*. Saintpaul	37 (13.9)
	*S*. Kentucky	20 (7.5)
	*S*. Typhimurium	15 (5.6)
	Others	70 (26.2)
Camels, (n = 116), [38]	*S*. Saintpaul	45 (38.8)
	*S*. Braenderup	26 (22.4)
	*S*. Muenchen	10 (8.6)
	*S*. Kottbus	7 (6)
	*S*. Havana	6 (5.2)
	Others	22 (19)

^‡^The data excludes isolates from holding pens and hand swabs [30].

The preponderances of the serovars differ from reports elsewhere. For instance, in the USA, *S*. Newport (48.71%), *S*. Agona (15.10%) and *S*. Typhymurium (7.07%) were the dominant isolates of bovine origin; *S*. Typhymurium (24.48%), *S*. Derby (14.72%) and *S*. Cholaraesuis (10.43%) were the three most common isolates of porcine origin [63] and *S*. Oranienburg (21.8%), *S*. Cerro (21.8%) and *S*. Anatum (10.3%) were the three most common isolates of beef cattle [42]. In Great Britain, *S*. Typhimurium (11.1%), *S*. Derby (6.3%) and *S*. Kedougou (0.9%) were the top three serovars isolated from pigs [39]. In Korea, *S*. Typhimurium (47.6%), *S*. Derby (20.6%) and *S*. Heidelberg (1.6%) were the three top ranking isolates from swine samples [44].

Whilst four serovars were dominant in two or more host species, eleven were dominant in only a single host. *S*. Eastbourne was dominant in cattle and pigs; *S*. Infantis was dominant in cattle and small ruminants; *S*. Saintpaul was dominant in pigs and camels and *S*. Typhimurium was dominant in cattle, small ruminants and pigs. The differences in the relative occurrences of the serovars by host species could be due to differences in host-serovar interactions. The genetic make-ups of hosts could affect *Salmonella* [64] and a serotype may have different capabilities to infect different hosts [65]. Breed differences in the humoral and cell-mediated responses of pigs against *S.* Typhimurium were also reported [66,67]. Moreover, environmental factors may influence the survival of serovars/strains and could possibly contribute to the within and between host species differences.

All serovars are generalists [68] and could be causes of outbreaks in humans and animals. Outbreaks associated with most of these serovars were recorded elsewhere. For instance, *S*. Braenderup, *S*. Infantis, *S*. Hadar, *S*. Heidelberg, *S*. Newport, *S*. Saintpaul , *S*. Typhimurium were causes of outbreaks that occurred between 2009 and 2014 in the USA [69]. Similarly, outbreaks due to *S*. Anatum in Japan [70], *S*. Kottbus in Spain [71], *S*. Eastbourne in Canada [72], *S*. Muenchen in Germany [73], *S*. Havana in Iran [74] and *S*. Kentucky in dairy cattle in the USA [75] were recorded. However, in Ethiopia, *S*. Typhimurium appears to be more important than others, because it was one of the dominant isolates in cattle, small ruminants and pigs (Table 4); it was isolated from camels [38] and animal products [57,59,60] and accounted for 9.4% of the total and 15.3% of the NTS isolates of human origin [18]. Globally, *S*. Typhimurium represents 10-30% of the human NTS isolates [76] and in SSA it is a common cause of invasive infection [16,77,78] with a high mortality in AIDS patients [79].

### Implications and limitations

A national survey on the prevalence of *Salmonella* in Ethiopia has not been carried out. However the present study highlights the prevalence of carrier animals and the pooled estimates could be used as inputs in re-enforcing the policy on meat safety in slaughter houses and butcheries and educating personnel in contact with animal products. Furthermore, regardless of the cultural taboos and the difficulties associated with the prohibition of backyard slaughtering, promoting educational campaigns to discourage the practice could reduce the risks of transmission of *Salmonella* from animals to man. In addition, although the economic stamina of the country and the infrastructure do not allow a nation-wide surveillance and monitoring of NTS, such systems and control measures could be implemented in high risk animal production systems (poultry, pigs and dairy cattle) because the number of such farms is very small.

Despite statistical evidences of heterogeneity, subgroup analyses were not done by potential risk factors but species. Moreover, pooled prevalence of *Salmonella* in dairy cattle and camels were not calculated because there are single reports on each. However, as the random effects model considers the studies as a sample of all potential studies, the estimates provide a relatively better picture of the occurrence of *Salmonella* and the comparative importance of food animals in Ethiopia.

## Conclusions

All food animals are considerable reservoirs of *Salmonella* and at least 15 serotypes appear to be of considerable concerns. The results justify the need for strict intervention measures to reduce contamination of carcasses in slaughterhouses and the transmission of *Salmonella* from animals to humans. Large scale studies are required to describe the epidemiology of the serotypes in the country.

## Additional file

Additional file 1:
**PRISMA check list.**
